# Herb medicine for relieving radiation induced oral mucositis: A systematic review and meta-analysis protocol

**DOI:** 10.1097/MD.0000000000018337

**Published:** 2019-12-16

**Authors:** Gui Wang, Liqun Jia

**Affiliations:** a Beijing University of Chinese Medicine; b Department of Oncology of Integrative Chinese and Western Medicine, China-Japan Friendship Hospital, Beijing, China.

**Keywords:** head and neck cancer, oral mucositis, protocol, radiotherapy, systematic review

## Abstract

**Background:**

Oral mucositis (OM) is a common and unavoidable side effect in patients suffering from head and neck cancer who are undergoing radiotherapy. It is characterized by unbearable pain, as well as eating and speech disorders. This has serious negative effects on the patients’ quality of life and can even reduce radiotherapy tolerance, ultimately resulting in a poor prognosis. At present, many prevention and treatment methods are still in the experimental stage, and the efficacies are controversial.

**Methods:**

Four English databases: Medline via pubmed, EMBASE, the Cochrane Library, Web of Science and another 4 Chinese databases: China National Knowledge Infrastructure (CNKI), China Science and Technology Journal database (VIP), Wanfang Database and CBM, will be searched from inception to August 2019. All randomized controlled trials in Chinese and English language will be included. Literature selection, data extraction and quality assessment will be completed by 2 independent authors. The primary outcomes will include the incidence of OM (1–4 grade) and the pain degree. The onset time of OM, the improvement rate for quality of life, and any adverse effects will be evaluated as the secondary outcomes. The data will be synthesized by Review Manager and Stata software.

**Results:**

This study provides a high-quality synthesis from existing evidence for Chinese herbal medicine in radiotherapy induced OM treatment, according to the criteria: incidence of OM, onset time of OM, status changes in quality of life and adverse events.

**Conclusion:**

This study will provide evidence to help determine whether Chinese herbal medicine is effective and safe for use in the prevention and/or treatment of radiotherapy induced OM.

**Ethics and dissemination:**

No additional formal ethical recognition or informed consent is required since no primary data collection is involved. The study result will be published in peer-reviewed journals or at related conferences.

PROSPERO registration number: PROSPERO CRD42019141900.

## 1 Introduction

Oral mucositis (OM) is defined as a disease characterized by inflammation or ulceration of the oral mucosa,^[1]^ and is one of the most common side effects of radiation therapy for patients with head and neck cancer (HNC), occurring in almost all patients.^[2,3]^ Radiotherapy-induced oral mucositis (RIOM) usually occurs 2 to 3 weeks after radiotherapy, and the pathobiology is complex and multifactorial. Put simply, radiation-induced DNA strand breakage of basal stem cells and clonogenic death prevents the renewal of superficial mucosal cells that normally shed. OM mainly presents with taste loss, xerostomia, and severe pain. This effects eating patterns and thus nutritional intake, which can lead to radiotherapy treatment interruption, then subsequently reducing the general health condition of the patient and their quality of life. Ultimately, this results in affecting the control of local lesions and prognosis adversely. Therefore, effective prevention and management of OM secondary radiation is very important. Currently, common clinical management strategies include standardized oral care, anti-inflammatory, antimicrobials, analgesics, growth factors, and topical agents. Most topical drugs are in the form of mouthwash, such as doxepin or benzydamine. Generally, it is composed of 2 or more kinds of anesthetics, antacids, diphenhydramine, nystatin, and dexamethasone. Benzydamine is a non-steroidal anti-inflammatory drug recommended in the Mucositis Study Group of the Multinational Association of Supportive Care in Cancer and International Society of Oral Oncology (MASCC/ISOO) Mucositis Guidelines, aiming to prevent the OM in HNC patients treated with medium-dose radiotherapy (up to 50 Gy), without chemotherapy. However, these drugs are mainly used to temporarily relieve pain symptoms, with limited effectiveness. Palifermin (keratinocyte growth factor-1) is the unique approved agent for mucositis prevention by the US Food and Drug Administration and the European Medicines Agency in patients with hematological malignancies treated by hematopoietic cell, but its high cost limits its widespread use.^[4]^ Low level laser therapy is another promising and effective method for reducing the severity of RIOM in patients with HNC who receive radiotherapy without concomitant chemotherapy, but its long-term safety and potential impact on tumor response remain unclear.^[5–8]^ According to statistics, the additional economic cost of RIOM treatment for each HNC patient is as high as $17,000 (though this is incomplete), which is a huge economic burden.^[9]^ Therefore, it is of great significance to find economical and effective treatment methods for RIOM.

Chinese herbal medicine (CHM) is a complementary alternative medicine which uses medical plants, minerals, and animal parts to prevent or treat disease. It originated in China and is popular all over the world.^[10]^ In recent years, more and more OM patients choose CHM to prevent or treat RIOM.^[11–15]^ The study shows that the herbal medicine of “clearing heat and detoxifying” type has anti-inflammatory and analgesic properties, and has a good effect in treating OM.^[12,16–18]^ CHM contains antioxidants that reduce the production of reactive oxygen species, thereby reducing mucositis.^[19]^However as far as we know, despite the increasing use of CHM, systematic reviews of CHM in the treatment of OM are limited to recurrent OM^[20–22]^ and pediatric OM.^[23]^ So far, there has been no systematic review on evaluating the effectiveness of CHM for relieving RIOM. Therefore, a comprehensive assessment of the efficacy and safety of herbal remedies for the prevention and treatment of RIOM will be conducted in this study to provide clinical evidence.

## 2 Methods

### 2.1 Study registration

The systematic review protocol has been registered on PROSPERO with the number CRD42019141900, link to the https://www.crd.york.ac.uk/prospero/display_record.php?ID=CRD42019141900. This protocol will follow the guidelines of Preferred Reporting Items for Systematic Reviews and Meta-Analyses Protocols (PRISMA-P).

### 2.2 Inclusion criteria for study selection

#### 2.2.1 Types of studies

All randomized controlled trials (RCTs) in Chinese and English language will be included. Uncontrolled trials, quasi-RCTs, case reports, case-controlled studies, animal studies, in vitro studies, qualitative studies, reviews and comments will be excluded.

#### 2.2.2 Types of participants

Patients of any age who have been pathologically diagnosed with head and neck malignant tumors who have undergone radiotherapy (with or without chemotherapy, targeted therapy or immunotherapy), who developed OM, will be included. According to the international agency for research on cancer (IRAC) definition of HNC in 2014, the main anatomical parts of the head and neck involving the nasal cavity, nasopharynx, oral cavity, oropharynx, larynx, and hypopharynx.

#### 2.2.3 Types of interventions

Trials using herbs medicine alone or herbs medicine in combination with conventional therapy will be included. Herb medicines includes single herb medicine, prescriptions composed of several herb medicines and herbal products extracted from the natural herbs. There is no restriction on the method, form, dosage or treatment time of administration. The control group received conventional treatment, no treatment or placebo. Conventional treatments include drugs such as antibiotics, analgesics and corticosteroids.

#### 2.2.4 Types of outcome measures

Primary outcomes

1. The incidence and severity of OM. Mucositis grades are defined according to world health organization (WHO). WHO criteria for mucositis on a 0 to 4 scale, where 0 is defined as none, 1 represents erythema or soreness, 2 indicates ulcer and able to eat, 3 is defined as ulcer and limited eating, and 4 indicates ulcer with hemorrhage and necrosis.
2. The pain degree of radiation-induced OM. The severity of oral pain will be evaluated based on a visual analog scale (VAS).^[24]^

Secondary outcomes

1. The onset time of OM, which was defined as the time of definitively diagnosed OM.
2. The improvement rate for quality of life.
3. Herbs medicine related adverse effect.

### 2.3 Search methods for identification of studies

#### 2.3.1 Electronic searches

We will extensively search studies in Chinese and English from the following databases from their inception to August 2019, including 4 English databases: Medline via pubmed, EMBASE, the Cochrane Library, Web of Science and another 4 Chinese databases: China National Knowledge Infrastructure (CNKI), China Science and Technology Journal database (VIP), Wanfang Database and CBM. Table 1 shows the search strategy of PUBMED database, which will then be translated into other languages and searched in the corresponding database.

**Table 1:**
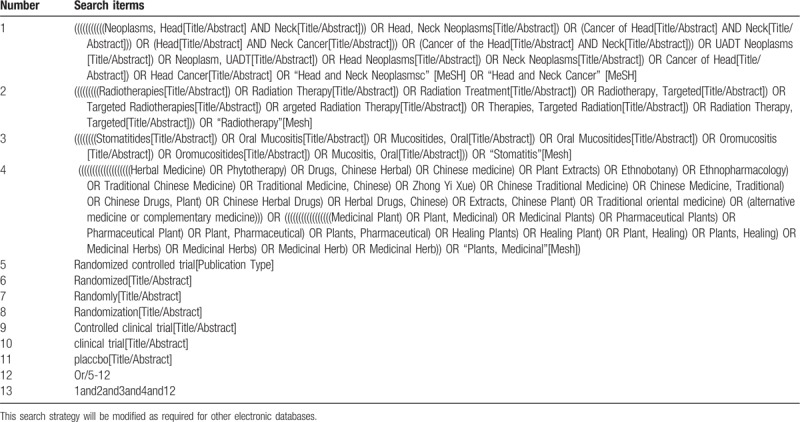
search strategy for Medline (via PubMed).

#### 2.3.2 Searching other resources

We will search the WHO international clinical trial registry platform (ICTRP) (http://apps.who.int/trialsearch/), PROSPERO, clinicaltrials.gov (http://www.clinicaltrials.gov) and metaRegister of Controlled Trials (mRCT; http://www.controlled-trials.com/mrct) for ongoing trials related to the topic. In addition, reference lists of previous published reviews, gray literature, relevant journals and conference abstracts will also be searched for eligible studies.

### 2.4 Data collection and analysis

#### 2.4.1 Selection of studies

**Figure 1:**
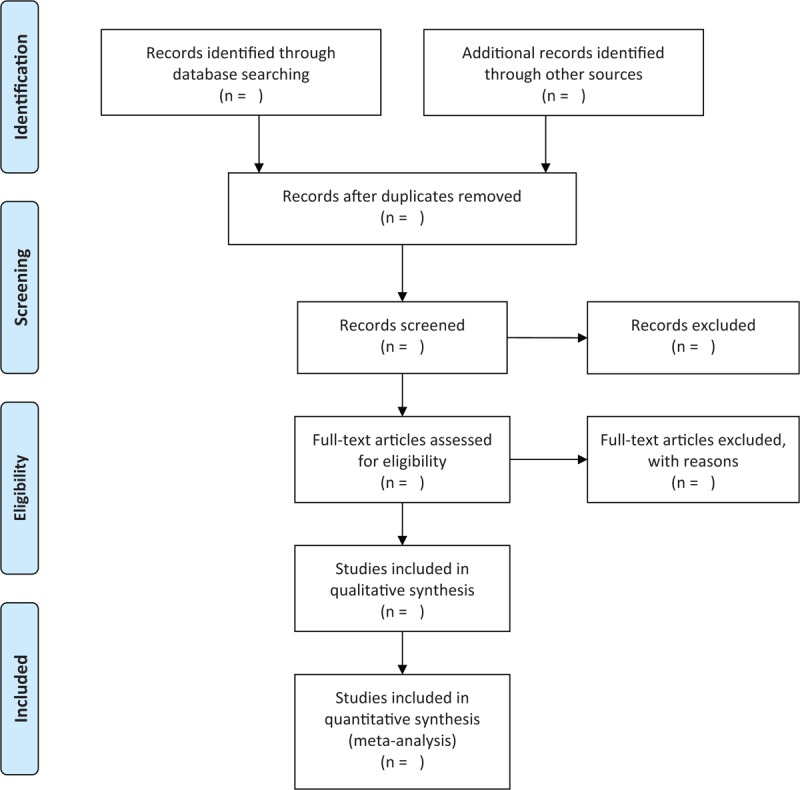
The PRISMA flow chart of the selection process.

The literature citations retrieved from the database will be imported into Endnote X8, which is to be used for literature management (such as deleting duplicate articles). The research articles that meet the inclusion criteria will then be screened by two researchers (GW, LQJ) independently, by scanning the titles and abstracts. The reasons for exclusions will then be recorded on standard eligibility forms. Studies in which two researchers’ judgments differ will be decided by discussing with a third researcher. The screening process is presented in the PRISMA flow chart (Fig. 1).

#### 2.4.2 Data extraction and management

A standard data extraction sheet based on the Cochrane Handbook for Systematic Reviews of Interventions guidelines will be developed, and two researchers (GW, LQJ) will independently extract data from the studies that meet the inclusion criteria. Information not clearly stated in article will be obtained through telephone or email contact from the first author. For each included study, the following data will be extracted:

1. Characteristics of the study such as first author, title, year, country, and sample size.
2. Characteristics of participant such as age, gender, number of patients included in analysis, type of cancer and details of radiotherapy.
3. Type of intervention (s) and comparator(s) with details such as dose, administration, and duration of therapy.
4. Outcome measures including definition of mucositis and time to measurement mucositis.

#### 2.4.3 Assessment of bias in the included studies

Bias will be assessed independently by 2 authors (GW, LQJ) based on Cochrane risk of bias (ROB) assessment tool.^[25–27]^ We will assess the risk of bias from the following aspects: random sequence generation, allocation hidden, blind method, incomplete result data, selective reporting of results and other deviations. We will classify each potential bias test as high, low, and unclear. If there are any outstanding differences, a third researcher will make the final decision as arbitrator.

#### 2.4.4 Measures of treatment effect

The risk ratio (RR) with 95% confidence interval (CI) will be used to analyze dichotomous data. For continuous data, standard or weighted mean differences (SMD or WMD) with 95% CIs will be used.

#### 2.4.5 Unit of analysis issues

To avoid the carry-over effect, we will only extract the data of the first experimental period of the crossover test. For the experiment of multiple intervention groups, all relevant control intervention groups and experimental interventions in the experiment will be combined into one group to avoid a unit-of-analysis error.

#### 2.4.6 Management with missing data

Any missing data will be retrieved by contacting the first author or other author of the article via phone or email. The article will be excluded if the missing data is still not available.

#### 2.4.7 Assessment of heterogeneity

Heterogeneity of the included studies will be detected by standard chi-square test and *I*^2^ statistics,^[28]^ and heterogeneity was demonstrated by visual forest map. In the chi-square test, *P* < .1 suggests that heterogeneity exists in the included studies. *I*^2^ is used to judge the degree of heterogeneity. The heterogeneity of the included studies is generally considered acceptable when *I*^2^ < 50%. When heterogeneity exists (*I*^2^ > 50%), sensitivity analysis and subgroup analysis were selected to detect the possible reasons for substantial heterogeneity.

#### 2.4.8 Assessment of reporting bias

Funnel plots were used to detect reporting bias when more than 10 studies were included.

#### 2.4.9 Data synthesis

The data will be synthesized by Review Manager and Stata software. A fixed effect model will be selected where multiple homogeneous studies are included, and a random effect model selected where studies with poor homogeneity are included. We will further analyze the causes of heterogeneity by subgroup analysis and meta-regression.

#### 2.4.10 Subgroup analysis

Where the number of studies is sufficient and a high heterogeneity exists, subgroup analysis will be conducted. Subgroup analysis on mucositis outcomes according to different administration patterns of herb medicine (such as oral administration or gargling) will be performed.

#### 2.4.11 Sensitivity analysis

We will evaluate the robustness and reliability of review results through sensitivity analysis. The sensitivity analysis will be based on methodological quality standards. A new meta-analysis will be conducted after excluding low-quality or non-blind studies, and the results and effects of the 2 meta-analyses will be compared and discussed.

## 3 Discussion

HNC accounts for 5% of all tumors, and is now the eighth most common cancer with about 710,000 new cases of these tumors diagnosed in 2018 global cancer statistics.^[29]^ The definition of HNC has not been confirmed worldwide, but according to the international agency for research on cancer (IRAC) includes the nasal cavity, nasopharynx, oral cavity, oropharynx, larynx, and hypopharynx. Of these, more than 550,000 new cases of specifically oropharyngeal, oral, laryngeal and hypopharyngeal cancers are reported worldwide each year. For the early stages (stage I or II), surgery or radiation therapy is recommended. However, for non-resectable diseases, radiotherapy combined with chemotherapy or cetuximab is the preferred treatment.^[30]^ Radiotherapy is an important means to treat HNC, however its toxic side effects should not be underestimated. OM is one of these side effects. It is very common, and exhibits dose-limiting toxicity. Clinical trial studies have shown that 60% of patients receiving standard radiotherapy also had severe OM, and 100% of patients receiving excessive radiotherapy had severe OM. The overall incidence of OM was 83%. Mucositis can cause excruciating pain, speech and eating difficulties, nutritional intake disorders, and poor treatment tolerance. This can lead to a significantly reduced quality of life and even interrupt the treatment, negatively affecting prognosis. In addition, severe oropharyngitis can occur, increasing the risk of infection and hospitalization as well as increasing economic expenditure.^[31,32]^ Many current treatment methods are still in the experimental stage and have not been accurately determined, therefore it is necessary to continue to explore alternative economical and effective treatment methods.

In recent years, herb medicines have been widely used in clinical trials of RIOM in HNC patients. Therefore, we will use a systematic review and meta-analysis to evaluate the efficacy and safety of herb medicines for the treatment of RIOM in HNC patients. However, this systematic review has several limitations. The quality of the included studies is low, and CHM interventions also vary from study to study. Due to the inconsistencies of the included studies, a high degree of heterogeneity may also exist. This is the first meta-analysis to evaluate the efficacy and safety of CHM in the prevention and treatment of RIOM in HNC patients. We expect that the review could provide a basis for herb medicines treatment of RIOM, and overall offer more options regarding treatments available to HNC patients suffering from RIOM.

## Author contributions

**Conceptualization:** Gui wang, Liqun Jia.

**Data curation:** Gui wang, Liqun Jia.

**Formal analysis:** Liqun Jia.

**Project administration:** Gui wang, Liqun Jia.

**Supervision:** Liqun Jia.

**Writing–original draft:** Gui wang.

**Writing–review & editing:** Liqun Jia.
